# Improving Digital Hospital Transformation: Development of an Outcomes-Based Infrastructure Maturity Assessment Framework

**DOI:** 10.2196/12465

**Published:** 2019-01-11

**Authors:** Patricia AH Williams, Brendan Lovelock, Tony Cabarrus, Marlon Harvey

**Affiliations:** 1 Flinders Digital Health Research Centre College of Science and Engineering Flinders University Adelaide Australia; 2 Cisco Systems Australia Melbourne, Victoria Australia; 3 Cisco Systems Toronto, ON Canada

**Keywords:** medical informatics, information infrastructures, digital hospitals, eHealth, implementation, capability maturity modelling, security

## Abstract

**Background:**

Digital transformation in health care is being driven by the need to improve quality, reduce costs, and enhance the patient experience of health care delivery. It does this through both the direct intervention of technology to create new diagnostic and treatment opportunities and also through the improved use of information to create more engaging and efficient care processes.

**Objective:**

In a modern digital hospital, improved clinical and business processes are often driven through enhancing the information flows that support them. To understand an organization’s ability to transform their information flows requires a clear understanding of the capabilities of an organization’s information technology infrastructure. To date, hospital facilities have been challenged by the absence of uniform ways of describing this infrastructure that would enable them to benchmark where they are and create a vision of where they would like to be. While there is an industry assessment measure for electronic medical record (EMR) adoption using the Healthcare Information and Management Systems Society Analytics EMR Adoption Model, there is no equivalent for assessing the infrastructure and associated technology capabilities for digital hospitals. Our aim is to fill this gap, as hospital administrators and clinicians need to know how and why to invest in information infrastructure to support health information technology that benefits patient safety and care.

**Methods:**

Based on an operational framework for the Capability Maturity Model, devised specifically for health care, we applied information use characteristics to define eight information systems maturity levels and associated technology infrastructure capabilities. These levels are mapped to user experiences to create a linkage between technology infrastructure and experience outcomes. Subsequently, specific technology capabilities are deconstructed to identify the technology features required to meet each maturity level.

**Results:**

The resulting assessment framework clearly defines 164 individual capabilities across the five technology domains and eight maturity levels for hospital infrastructure. These level-dependent capabilities characterize the ability of the hospital’s information infrastructure to support the business of digital hospitals including clinical and administrative requirements. Further, it allows the addition of a scoring calculation for each capability, domain, and the overall infrastructure, and it identifies critical requirements to meet each of the maturity levels.

**Conclusions:**

This new Infrastructure Maturity Assessment framework will allow digital hospitals to assess the maturity of their infrastructure in terms of their digital transformation aligning to business outcomes and supporting the desired level of clinical and operational competency. It provides the ability to establish an international benchmark of hospital infrastructure performance, while identifying weaknesses in current infrastructure capability. Further, it provides a business case justification through increased functionality and a roadmap for subsequent digital transformation while moving from one maturity level to the next. As such, this framework will encourage and guide information-driven, digital transformation in health care.

## Introduction

### Background

Digital transformation in health care is being driven by the need to improve quality and reduce costs in health care delivery, while taking advantage of the benefits that advances in emerging technology can provide to patient care, the patient experience, workforce performance, and value and efficiency in health care delivery [1,2]. The recognition that digital transformation is important to health care is reflected in increasing discussions on what the digital health care future looks like and how we are moving to virtualized care venues, smart monitoring, and new trials using the Internet of things and cloud services [2,3]. Several studies have indicated that specific clinical initiatives require the improved support of health information technology infrastructure [4,5]. However, it is not the digital transformation itself that is the goal, but the capabilities and advances that this transformation can realize. While the fee-for-service health care funding model has been predominant in many countries, particularly outside of public hospitals, there is a shift to value-based health care funding with payment based on patient health outcomes. Hence, this shift necessitates assessment of the infrastructure to support new technologies and how the infrastructure aligns to clinical and administrative business value. Indeed, digital transformation will be critical to the survival of health care organizations [6].

To take advantage of technologies that can improve information flow in the digital hospital environment and enhance processes to create a positive impact on both clinical and operational outcomes, an assessment of the capabilities of the existing environment is required. Only through this process can we create a map for improvement and possibilities. Currently, there is no language to describe hospital technology infrastructure and no accepted standard methodology to assess the state of the network and infrastructure in a hospital to support structured improvement aligned to business processes. While there is such an industry assessment measure for electronic medical record (EMR) adoption using the Healthcare Information and Management Systems Society (HIMSS) Analytics EMR Adoption Model (EMRAM) [7], there is no equivalent for assessing the infrastructure and associated technology capabilities for digital hospitals. An infrastructure assessment framework is important to fill this gap as hospital administrators and clinicians need to know how and why to invest to support information infrastructure for the future. Such a framework will encourage and guide information-driven, digital transformation in health care.

This paper defines what information infrastructure a digital hospital requires to mature and use technology to enable effective information flow and support the clinical and patient experience, now and in the future. The articulation and development process leading to the infrastructure maturity model and the information characteristics required to achieve the maturity objectives are given in the following section. This includes the mapping of systems capabilities to meet clinical process and patient experience, followed by a description of how we used a health care–specific capability mapping tool to devise a practical and industry best-practice assessment and scoring tool. The results demonstrate how this capability assessment mapping can be used to assess the maturity of a digital hospital to meet and improve its capabilities and how such a tool can be used for future planning of digital hospital technology requirements aligned to business and clinical outcomes.

### Defining Information Maturity in Health Care

This section describes the reasoning behind, and formulation of, the maturity levels used to define infrastructure maturity, as well as the dimensions of information needed to place “information” in a position of enabling and supporting health care processes and decision making. It details the technology and technology services required to achieve those information dimensions and how those capabilities are aggregated into a set of naturally evolving levels. This description includes the research process of deconstruction of the associated technology requirements into a framework to determine the maturity of digital hospital infrastructure.

### Information Dimensions to Enable and Support the Health Care Process

“Enabling information” means information that intrinsically possesses qualities to ensure it contributes effectively to health care delivery and decision making. This refers to both administrative and clinical data. The characteristics of enabling information in health care are analogous with data quality and comprise similar elements. Indeed, “information quality plays an important role as a mediator between clinical information technology and health care quality” [8]. Further, in different contexts, data quality may mean different things. For instance, administrative data are used both within the hospital environment for planning and funding, and also by the managing jurisdictions for services planning and policy making. Routine hospital data used outside the organization require standardization and homogenization for comparison within and across jurisdictions [9]. Further, data of lower quality can significantly influence business processes as well as clinical care [10]. A range of characteristics define information features and qualities that support patient care and its associated workflow processes [11]; this is a multidimensional concept [8]. Russ et al [11] categorize such data quality characteristics into four domains: trustworthy and reliable, effectively displayed, adaptable to work demand, and ubiquitous. Each of these domains further defines the data characteristics that support that domain. For instance, the trustworthy and reliable domain consists of the data characteristics complete, consistent, correct, current, and secure. These characteristics are closely aligned to data quality characteristics.

In the context of health care, there are numerous definitions of what core quality characteristics should consist of, including accuracy, completeness, currency, and consistency, yet the associated characteristics of reliability, availability, usability, relevancy, and secureness are also important, in addition to trust, usefulness, and redundancy [10,12-16]. All these characteristics are accepted quality metrics throughout the literature. This paper does not, however, present a discourse on the ontology of data quality characteristics but instead accepts the premise that such characteristics exist and form the basis for extrapolation of the use of data with such embedded characteristics. These embedded characteristics provide the foundation for enabling information in health care.

There is an overlap between many of the data qualities. Therefore, each quality cannot be considered in isolation as a discrete concept because qualities come together in the context of use rather than through definitional articulation. There are numerous qualities that can describe data and information; however, in the health care environment these are not orthogonal or mutually exclusive. The selection of the following six higher-level characteristics used in this research reflects the usability of such characteristics when applied to health care:

1. Completeness: the information contains all the context required for decision making
2. Relevancy: the information required for the task at hand
3. Usability: information delivered on/in an appropriate device/format
4. Availability: information is available where it is required and exists in accessible systems
5. Reliability: information is sufficiently complete, error free, and consistent in distributed settings
6. Security: the information process is protected against extraction or tampering

These six characteristics give an insight at a high level into the importance of information in clinical application and are consistent with research into the use of patient information in electronic form [11] and how this influences operational and clinical workflow and service delivery. Further, each of the six dimensions have multiple interdependencies (see Table 1). Table 1 is based on the work of del Pilar and Garcia-Ugalde [14] with correlation from Russ et al [11] and Lee et al quality assessment characteristics [17].

A systematic review by Lee et al [17] identified accuracy as an intrinsic information quality across all studies analyzed. Using accuracy as one characteristic to explain these interdependencies, accuracy appears as a component of several dimensions but not one of the primary data quality dimensions in Table 1, because while accurate information is needed, on its own it does not enable health care delivery. However, accuracy is a primary element of the dimension of relevancy. Relevance refers to the information being appropriate for the task at hand, and information cannot be appropriate without being suitably accurate.

The interdependence of dimensions, such as accuracy and completeness (another intrinsic element that applies to multiple dimensions), demonstrates that attempting to definitively separate the dimensions is problematic and not productive. It should be noted that the dimension of “security” is a special case that supports all other dimensions.

The data quality dimensions (ie, characteristics of usable information) described in Table 1 are applied in this research to structure a scale of information maturity levels in an organization. They provide a loose set of guiding principles on how a hospital improves its use of information as it digitally matures. The characteristics are linked to the hospitals’ supporting information technology and technology services through the Capability Maturity Modeling (CMM) process described in the following section.

**Table 1:** Data quality dependencies adapted from [14].

Data quality dimension	Interdependencies
Completeness	Coverage, density, relevancy, and sufficiency
Relevancy	Current, timely, correct, and sufficient
Usability	Usefulness consisting of relevance accuracy and completeness Easy to use and organized
Availability	Accessible, compatible, interpretable, and locatable
Reliability	Unbiased Reputation traceability including data source and provenance Data producer with previous experience and correction of mistakes Credibility inclusive of accuracy and completeness Consistency
Security	Supports all other dimensions

### Capability Maturity Model

In the 1980s, CMM was devised as a tool to assess internal and external improvement processes [18], enabling a transformation from chaotic and ad hoc process implementation to definitive and disciplined process execution. CMM has an established background in software engineering, as well as applications to user-centered design [19], education [20], and information systems planning [21], for example.

The CMM methodology is based on ongoing improvement of capability and is constructed using:

- Maturity levels. These provide a structured template for persistent improvement. They promote the ability to assess new practice implication and success.
- Key process areas and their associated goals. A key process area is a set of related activities that can achieve the stated goal for that key process area.
- Common features. These are categories of key process areas and reflect the effectiveness and repeatability of that key process area. The CMM common features are commitment to performing, ability to perform, activities performed, measurement and analysis, and verifying implementation.
- Key practices. The implementation and persistent achievement in a key process area are defined by the procedures, practices, and communications implemented called key practices [22].

**Figure 1:**
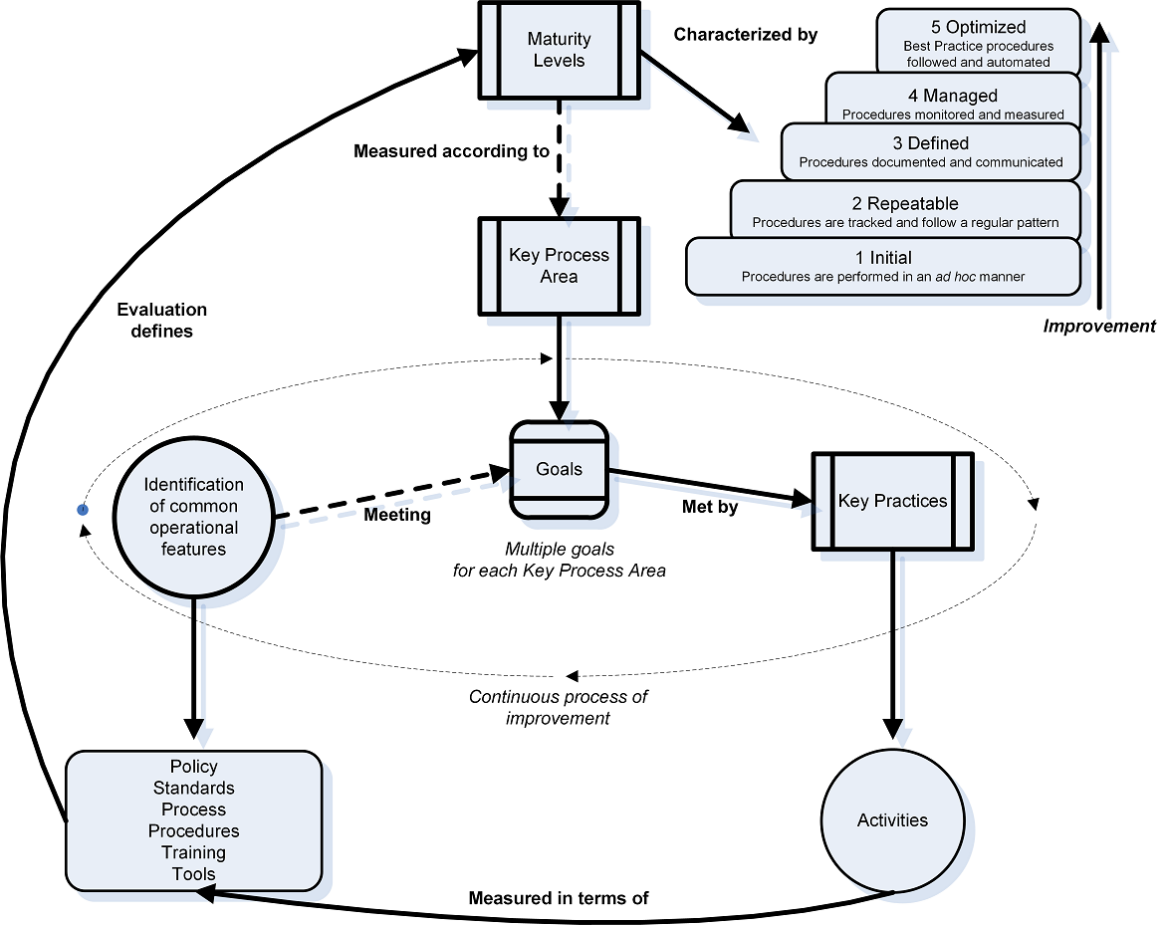
Representation of Capability Maturity Model as an operational framework.

The adaptation of this CMM methodology into an operational framework (Figure 1) that can assess security technology, process, and human contribution in a medical environment in a simple and straightforward manner was developed by Williams [22].

This adaptation enables us to assess capability, competency, and maturity as a development of function, through the construction of a matrix of capability against competency that defines the maturity level reached (Table 2). The operational CMM framework (Figure 1) has been successfully applied to practical cybersecurity assessment of primary health care in Australia [23]. Table 2 is an example of this application in Australian primary health care, using back-up activities to articulate the key practice of back-up within the key process area of business continuity. As demonstrated in Table 2, the levels (1-5) define increasing competency in specific, deconstructed back-up activities.

A comprehensive discussion on the development of this framework can be found in Williams [22]. The framework was adapted to the context of this research as it relates to digital hospital infrastructure (described in the Methods section).

### Enabling Information Mapping Using Capability Maturity Modeling

**Figure 2:**
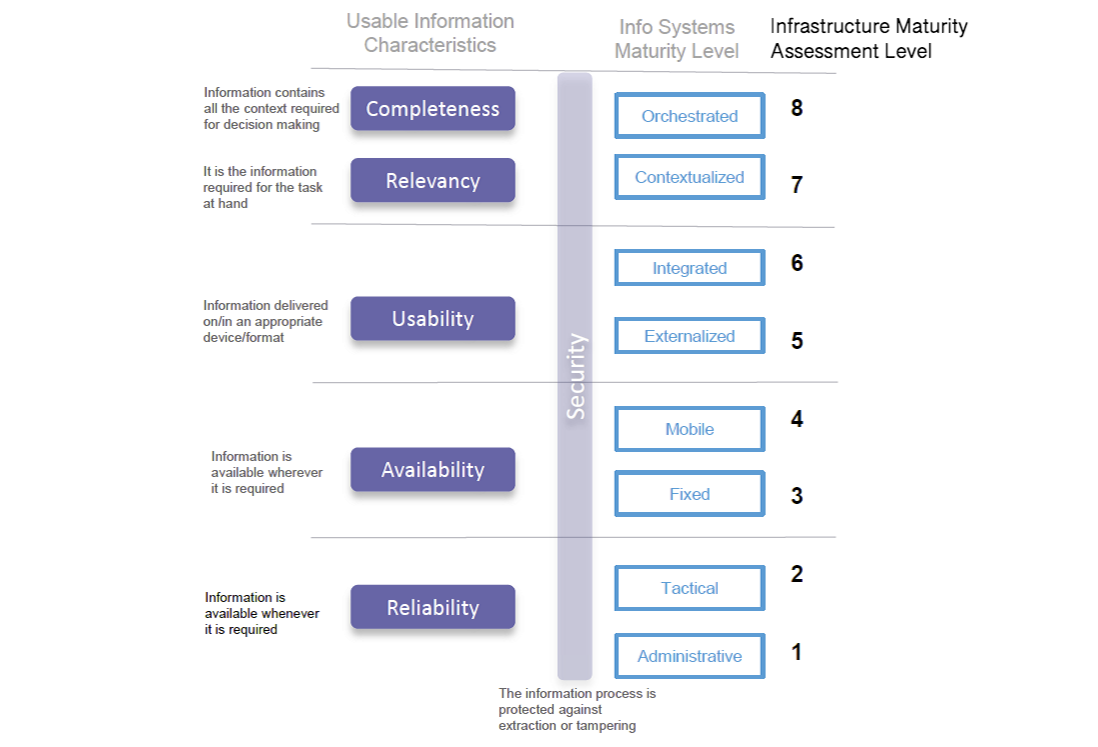
Information systems Infrastructure Maturity Model.

The HIMSS EMRAM maturity model, which measures the degree to which hospitals have replaced paper-based processes with technology, omits the ability to understand the supporting information and communication infrastructure required to achieve each level of maturity. The HIMSS EMRAM maturity levels were used as the starting point from which to devise the capabilities required to support digital technologies to deliver optimal operational experience and business value. Using the principles of CMM, an eight-level (rather than the traditional five-level CMM) information systems Infrastructure Maturity Model (IMM) was developed (Figure 2). This was developed using Cisco Systems Australia’s extensive experience in designing and building hospital infrastructure, by articulating the definition of infrastructure capability to support key domains, clinical services, and the application stack required to meet models such as HIMSS EMRAM. This was further enhanced through a series of observational ethnographic studies of hospital emergency departments that investigated how hospitals link the use of information with information technology and services that support a hospital’s information-driven processes. These studies looked at the way technology is integrated into the clinical process, from simple to complex, with reference to the HIMSS EMRAM structure and level of sophistication required at each layer [24].

The IMM provides a framework for determining the preparedness of a hospital facility to support existing and planned process rollouts of digital infrastructure by classifying the way hospitals manage their digital infrastructure and by reflecting the sophistication of the information processes used within the organization. Each of the maturity levels is characterized in terms of the experiences they generate for key stakeholders (Table 3). The expected stakeholder experience at each of the maturity levels incorporates the patient experience, clinical process and quality, and associated productivity outcomes at a business level. This was tested, refined, and socialized with hospital Chief Information Officers through Cisco contacts to ascertain its relevancy and applicability.

Subsequently, the technology features and services (systems capability) for each maturity level were identified from the stakeholder experiences. These features demonstrate how technology can facilitate increased sophistication in health care delivery and the expected experience, reflecting the integration of technology and services (Table 4).

### Summary

**Figure 3:**
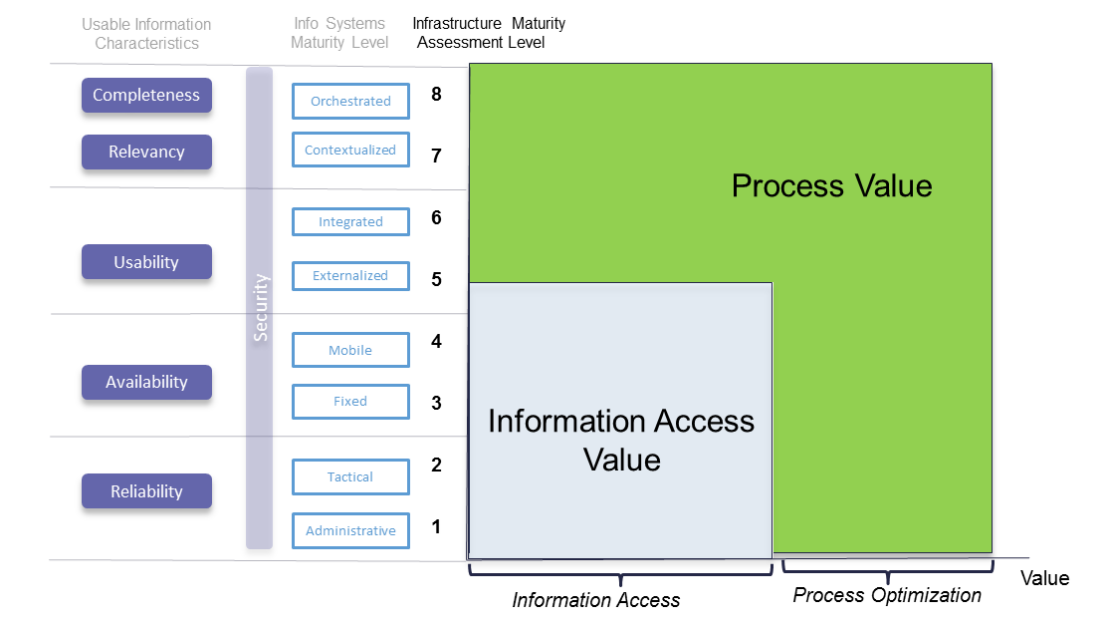
Value outcomes of infrastructure maturity.

To demonstrate the business value of the IMM, Figure 3 shows how the initial five levels increase information access. Once the five levels have been achieved, the value can be seen through process and business improvement.

It is important to make the distinction between access value and process value. Improving the ability to access information enables the potential to create new and improved processes. However, these processes may not be realized because the supporting infrastructure that enables the application of this information may not exist. Articulation of the IMM into the Infrastructure Maturity Assessment (IMA) defines the outcomes a hospital could aspire to (ie, Levels 6-8) from enabling full utilization of the information accessed in Levels 1-5.

**Table 2:** Extract of operational Capability Maturity Modeling (CMM) matrix for back-up capability [22].

Back-up capability (activities)	Level 1 Initial	Level 2 Repeatable	Level 3 Defined	Level 4 Managed	Level 5 Optimized
Back-up frequency	None or manual initiation on ad hoc basis, or unknown	Manual initiation ad hoc, weekly, or every few days	Manual initiation daily	Automatic initiation daily	Automatic initiation. Continuous/real time with checks in place
Back-up type	None or partial (data only) or incremental	Partial (data and set-up files)	Full – all data	Full – all data and programs	Full systems back-up or imaging, including operating system
Back-up encryption	None	None	Encrypted	Encrypted with password	All back-ups encrypted and password-protected. Appropriate password protection control
Back-up reliability	None or back-up not checked, or unknown	Back-up checked for completion	Back-up periodically checked for reliability	Back-up periodically checked for reliability and outcome tracked	Back-up reliability tested with automatic notification. Every back-up outcome tracked

**Table 3:** Extract of stakeholder experience for the eight levels of data use maturity.

Maturity level for data use	Stakeholder experience description
Level 8: Orchestrated	The clinical and patient experiences can be molded not only to the role of the person but to their location, who is around them, and the requirements of the individual clinician or patient. The patient can be dynamically guided to where their next appointment is, advised if the appointment is running late, and prompted just before the doctor is ready to see them. They can be delivered educational material at the most appropriate time as well as advice on support services they may need as they exit the hospital. These types of services can come to their bedside terminal if they are a patient or to their personal phone if they are an inpatient. The same types of customized services can be delivered to clinical and operational staff in the hospital, enabling them to better manage their tasks and access the most important information or people they require for the task at hand.
Level 7: Contextualized	The clinical information is now customized to specific roles. There is a high level of data interoperability between clinical systems, and clinicians can get a single pane view of the patient. Task management and alerts are available and implemented according to operational and model of care requirements. Task management and alerts are closed loop, that is, there are escalation paths when tasks and alerts are not appropriately processed. Tasks and alerts are sent directly to the required individual’s mobile device rather than to their desktop. Patients can access information at their bedside terminal, which is customized to the individual patient’s needs. This includes building services such as catering, lighting, temperature, and other support services. Patient and staff needs can be centrally monitored and support delivered as required either from the nursing station or a centralized service delivery hub.
Level 2: Tactical	The hospital is starting to use information technology for clinical purposes. They have several clinical applications that are not linked (typically patient administration system [PAS], pharmacy, pathology, and radiology), and the network has sufficient speed to support these applications where they are required. There is a recognition of the importance of their PAS, and there are robust disaster recovery processes in place. The clinical applications are not always available to the clinical staff. Ordering results and general reporting are via paper and forms. The PAS system provides the central information resource. The information from the PAS is limited to a restricted number of operational and clinical staff. The requirements of the biometric devices in the facility have driven the deployment of data grade wireless where it is clinically required. The voice communications process is seen as an increasingly important element of clinical collaboration, and there is basic Internet Protocol telephony with a full featured console.
Level 1: Administrative	Hospitals do not use information technology for clinical use in any significant fashion. They do use information technologies for operational and financial purposes. These hospitals are paper-based in their clinical processes. They use fax, mail, and desk phones for communication and collaboration. Ordering and reporting are via forms. Information retrieval is via paper patient notes and internal paper courier services.

**Table 4:** Technology features and services in the Infrastructure Maturity Model (IMM).

Maturity level	Data use	Technology services
Level 8	Orchestrated	Ability to link and coordinate processes in a centralized and automated fashion Agile infrastructure, adaptable to the changing needs of the facility
Level 7	Contextualized	Clinical processes customized to role and context Closed loop alerts and tasks Patient, staff, physical devices, and other resource location identification Analytics and dynamic resource management
Level 6	Integrated	Clinical processes on mobile devices Combined info views for staff and patients Bring your own device for staff and patients Building Management Systems integration Location services for key staff
Level 5	Externalized	Ability to virtualize the major clinical and operational hospital services for delivery independent of location
Level 4	Mobile	Clinical data available on mobile devices Widely used mobile voice communications Video services where needed High level of collaboration services Intelligent patient services Duress services widely available Locations services for equipment
Level 3	Fixed	Broad digital clinical data availability Ordering and reporting largely paper Results online, clinical data repository Integrated and distributed telephony services High performance personal computers
Level 2	Tactical	Department level apps to selected staff Ordering/reporting/accessing are paper-based Centralized high-quality telephony services
Level 1	Administrative	Limited clinical applications Paper-based systems Analogue voice communications

The characterization of a hospital’s infrastructure maturity using such a framework enables (1) identification of weaknesses in Information and Communications Technology (ICT) infrastructure capability, (2) definition of a business case justification of ICT investment, (3) provision of a roadmap for digital transformation in health care, and (4) measurement of international benchmarking of hospital infrastructure performance.

The usefulness of such a structure for characterizing health care ICT comes from the ability to link maturity levels and experience statements with the technology and services that need to be in place to deliver them. This research addresses the gap in the articulation of this technology infrastructure and services into practical and measurable capabilities.

## Methods

**Figure 4:**
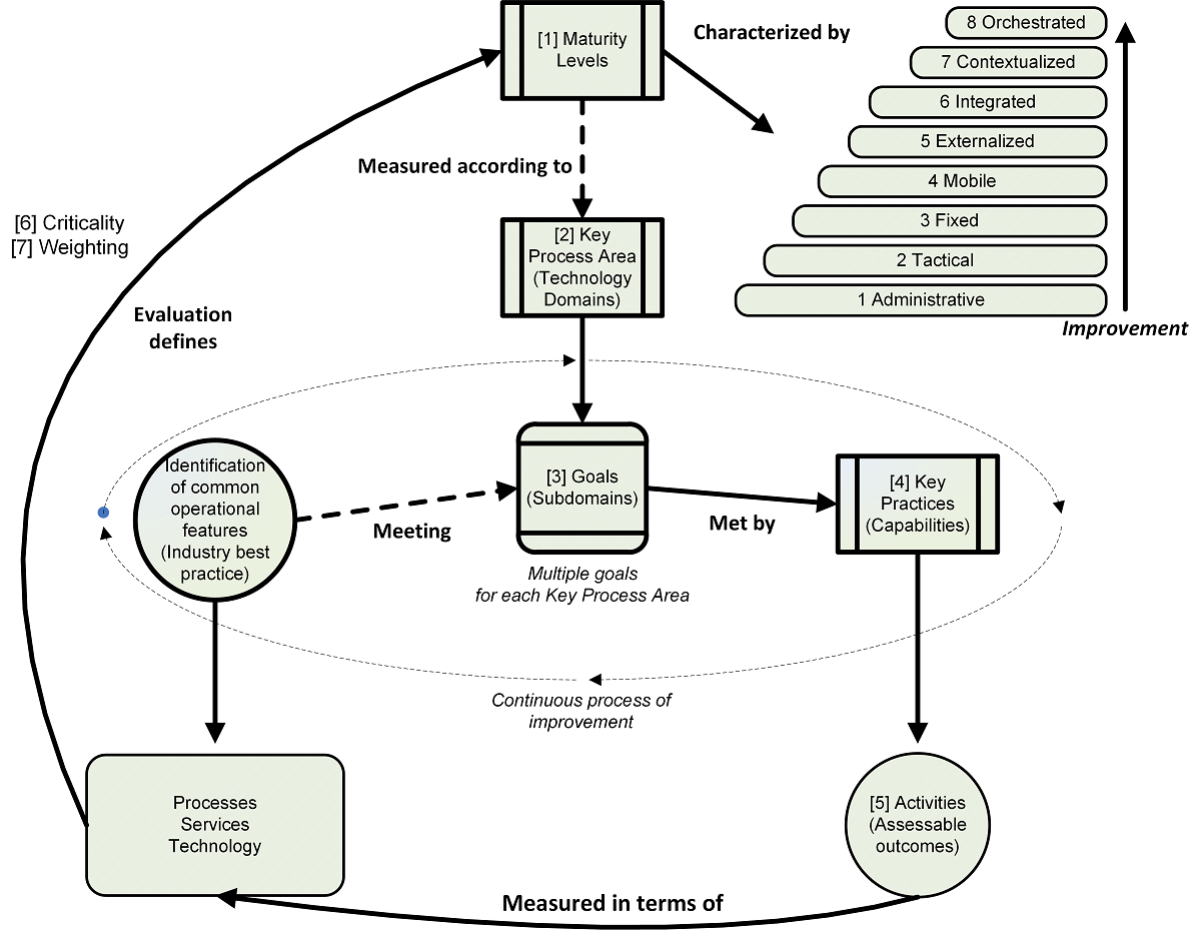
Contextualized operational Capability Maturity Model for infrastructure maturity assessment.

In order to translate the requirements into assessable capabilities, we used the Williams Operational CMM Framework (Figure 1), by contextualizing it for this research (Figure 4). The adaptation consisted of extending the number of maturity levels from 5 to 8, numbering the boxes and paths to reflect process steps below, and adding scoring components (6 Criticality and 7 Weighting). We also added text for correlated infrastructure terminology: Key Process Areas became (box 2) Key Process Areas (technology domains), Goals became (box 3) Goals (subdomains), Key Practices became (box 4) Key Practices (capabilities), and Activities became (box 5) Activities (assessable outcomes).

Using this framework (Figure 4), the process steps to create an operational infrastructure maturity assessment tool were as follows:

- Step 1 Maturity Levels: Define maturity levels, which means identifying and defining maturity levels appropriate to the target context (as described in Table 3).
- Step 2 Key Process Areas (Technology Domains): Identify key process areas (technology domains) means identifying the technology domains needed to support the outcomes for each of the maturity levels (from Step 1).
- Step 3 Goals (Subdomain Functions): Generate goals (subdomain function) comprises deconstructing each domain (from Step 2) into distinct, although not necessarily discrete functions, of that domain, called subdomains.
- Step 4 Key Practices (Capabilities): Devise key practices (capabilities) involves deconstructing each subdomain (from Step 3) into capabilities expected to meet each goal (subdomain function).
- Step 5 Activities (Measurable Outcomes): Articulate activities (measurable outcomes) means articulating measurable outcomes for each key practice capability (from Step 4) for each maturity level. The outcomes of this step also specify improvement from one maturity level to the next. This is represented as a capability matrix.

To facilitate a numeric scoring calculation of assessed infrastructure maturity, additional steps for assessment were devised.

- Step 6 Critical/Noncritical Capabilities: Identify the critical and noncritical capabilities for each key practice (from Step 4).
- Step 7 Assign Weightings: Assign weightings of importance to the goal subdomain (from Step 3) for each capability (from Step 4).

The research team consisted of the researcher, two Cisco infrastructure engineers and a health care technology expert, all with extensive experience in health care and the hospital environment in Australia and the United States. The construction of the framework was purposely constructed as nonproprietary and therefore industry generalizable. All capabilities researched and found to be Cisco specific were omitted from the capabilities. These capabilities may be explored for future revisions of the framework and for driving future technical innovation.

## Results

### Step 1: Maturity Levels

Eight maturity levels were identified and defined as appropriate to the digital hospital context. This definition and underlying reasoning for the eight levels of infrastructure maturity are explained in Table 3.

### Step 2: Key Process Areas (Technology Domains)

In defining the technology landscape to articulate the key process areas (technology domains), existing categories of outcome-based functionality were apparent. To manage the large scale of a hospital landscape, it was logical to group the technology and services into existing categories of outcomes-based functionality. These categories reflect five technology domains: (1) Transport, (2) Collaboration, (3) Security, (4) Mobility, and (5) Data Center.

### Step 3: Goals (Subdomain Functions)

The subdomains are the division of each domain into distinct, although not necessarily discrete (for that domain), functions of that domain. The methodology, by definition, looks at the delivery of services through infrastructure and therefore includes some services as infrastructure.

To illustrate the results of this methodology, the subdomain, “Transport” is used as an example. In the Transport domain, six specific functions (subdomains) of transport in infrastructure were identified: (1) Campus Connectivity, (2) Secure Remote Access, (3) Traffic Optimization–Quality of Service (QoS), (4) Disruption Tolerance and High Availability, (5) Management, and (6) Extensibility.

### Step 4: Key Practices (Capabilities)

The technology subdomains are further subdivided into key practices called capabilities. Each capability is described by a set of related information systems outcomes. The technology and technology services required to deliver those outcomes are then sequenced from Levels 1-8 of the IMA framework. Table 5 is an extract from the Transport domain using the Campus Connectivity subdomain and three of the capabilities from this subdomain together with the measures of capability (activities) at each maturity level. Levels 1 and 8 are presented as examples, together with the abbreviated descriptor of each capability.

**Table 5:** Extract of Transport domain, Campus Connectivity subdomain, with capability descriptors and measurable outcomes.

Capability	Descriptor (abbreviated)	Level 1^a^	Level 8
Cabling standard	ANSI/TIA-1179-A “Healthcare Facility Telecommunications Infrastructure” specifies recognized cabling and cabling category recommendations for health care facilities.	Category 6A cabling is ≤30%	Category 6A cabling >91%
Virtualization	The concept of virtualization applies tags to network packets that create the appearance and functionality of network traffic that is physically on a single network but acts as if it is split between separate networks.	No layer 2/3 virtualization implemented	Access controlled, policy-based micro segmentation of campus infrastructure based on virtual extensible local area network
Access port design and policy	A well-defined access port policy is based on the requirements of the end devices and the access of the applications and services by that end device.	No access port policy	Software defined automation access port configuration per software defined networking (SDN) policy
SDN integration	The SDN controller should support integration using application programming interfaces (APIs). Representational State Transfer (REST) APIs enable automation, integration, and innovation. All controller functionality should be exposed through these REST APIs.	No SDN	Demonstration of SDN contextual workflow using API integration

^a^Levels 2-7 (no entries) define increasing maturity assessment criteria.

### Step 5: Activities (Measurable Outcomes)

The activities (Table 5) express measurable outcomes (technology/technology services) for each capability at each maturity level, thus creating a capability matrix. In doing so, this matrix also specifies improvement from one maturity level to the next, which may be through increasing functionality, quality, or provision of a technology/service. The placement of each measurable outcome to a maturity level is based on best practice, in-depth knowledge, and experience by the Cisco design and implementation engineers and the external contributor to the project with expertise in health care technologies, networking, and CMM in the health care environment.

### Step 6: Critical / Noncritical Capabilities

As demonstrated throughout this paper, a digital hospital’s infrastructure is complex with many facets. To provide a collated assessment of the maturity to meet the business goals, a measure of essential and nonessential capability is required. Once these capability measures were defined, the critical and noncritical capabilities were identified for each capability within a domain and subdomain. These decisions were based on the importance of a specific capability to meet the business outcomes at that maturity level and the risk to the associated service delivery in not achieving this capability at this level. The critical capabilities are defined as essential criteria and a requirement that hospital facilities must meet to be placed at this level, regardless of other components reached at that level. If a capability is noncritical, then it is desirable but not essential for the maturity level.

### Step 7: Assign Weightings

Weightings are also assigned to each capability reflecting the importance of the capability within the subdomain. This is in addition to the critical and noncritical status of the capability because the criticality measure is a binary measure, and where there is more than one critical and noncritical capability, their importance to the subdomain is not necessarily equal. This allows for the more granular calculation of weighting specifically related to the maturity levels and the expectations in a hospital environment of what that maturity level would allow the organization to undertake both clinically and administratively.

### Summary

The collective set of matrices created using this methodology make up the IMA framework. Across the framework, there are a total of five domains, 34 subdomains, and 164 individual capabilities defined (see Multimedia Appendix 1).

## Discussion

### Principal Considerations

This research resulted in the identification of five key process area technology domains (Transport, Collaboration, Security, Mobility, and Data Center) each comprising multiple distinct subdomains. The 34 subdomains define functionality spread across the five technology domains. The articulation of this functionality into 164 measurable capabilities was achieved through the definition of each capability at each of the eight maturity levels.

We anticipate that the framework would be used to guide organizations as they go through major digital transformations. These transformations can be driven by the implementation of major clinical systems (such as EMRs), the refurbishment of brownfield facilities, or the building of new hospitals or area health services.

The assessment of capabilities is an uncomplicated exercise to match current practice to the best-fit maturity level, although technical knowledge of the implemented infrastructure environment is required. Each domain/subdomain identifies the capabilities that need to be in place. The measurable outcomes specify in detail how this is undertaken, reflecting the competency and maturity levels.

The challenges in defining and deconstructing each domain and its capability included a common understanding of terminology from a technical perspective and expansion of this for a broader technology informed audience. In developing a capabilities matrix, particularly one with the necessary eight levels, the challenge is that some capabilities may not change across each individual level. Therefore, assigning a level for that capability and subsequently calculating its weighting can be problematic. Where this occurs in the assessment, the highest level that capability can be assigned is allocated. This does not impact the weighting calculation for the level attained because the highest level is fundamentally determined by whether the critical capabilities for the domain or subdomain are met.

The process of using the IMA framework consists of (1) analysis of the hospital’s information systems infrastructure across the five technology domains, (2) classification matched to the eight-level maturity model against the relevant operational outcomes, (3) a roadmap and tailored objectives for each technology area outlining efforts needed to improve capability, (4) comparison and benchmarking of a digital hospital’s information infrastructure capabilities, and (5) recommendations for achieving business and IT goals to meet business and experience outcomes.

### Infrastructure Maturity Assessment Outputs

The following examples demonstrate typical outputs of the IMA framework and how they could be used by C-Suite executives (Chief Executive Officer, Chief Information Officer, Chief Marketing Officer, Chief Medical Informatics Officer) as a reference point for decision making on infrastructure investment.

The IMA output demonstrates the ability of the framework to create a benchmark for measuring the capabilities of a given hospital or health service. Further breaking the result down into technology subdomains and capabilities provides a detailed fingerprint of the ICT capabilities and how they relate to the requirements and aspirations of the health care provider.

**Figure 5:**
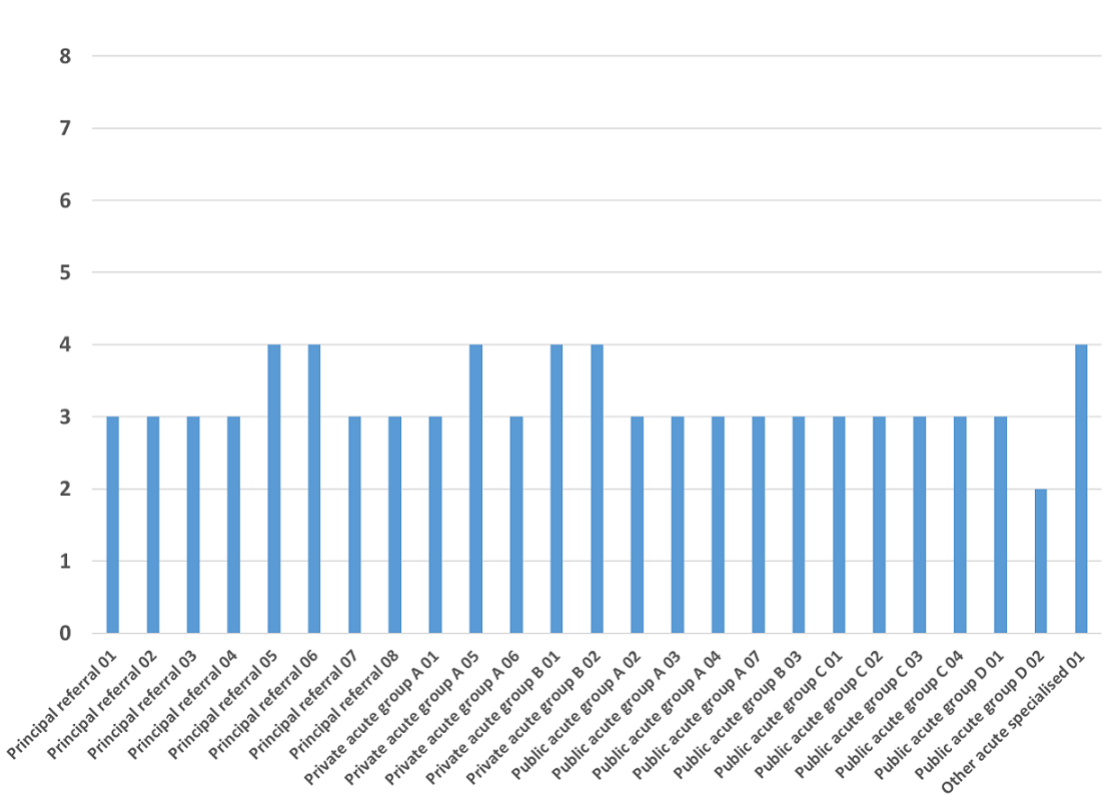
Example of the overall maturity assessment measure across 25 hospitals in Australia.

Figure 5 provides a picture of the current state of maturity across 25 de-identified Australian hospitals and demonstrates that most hospitals are operating at Level 3 (Fixed) infrastructure maturity. This level of capability creates significant limitations in the ability of the hospital facility to take advantage of technologies aimed at improving information flow and process operations within the hospital facility through achieving Level 4 (Mobile).

Level 3 describes an organization with limitations in its wireless, transport, and collaboration capability, restricting the ability to reliably access, share, and act on clinical information throughout the facility. These limitations diminish the value of the information applications that the organization has invested in by restricting the availability of information to when the clinical staff have access to personal computer endpoints. Ultimately, this limits the ability to drive clinical and operational process automation that can be gained from making data mobile.

**Figure 6:**
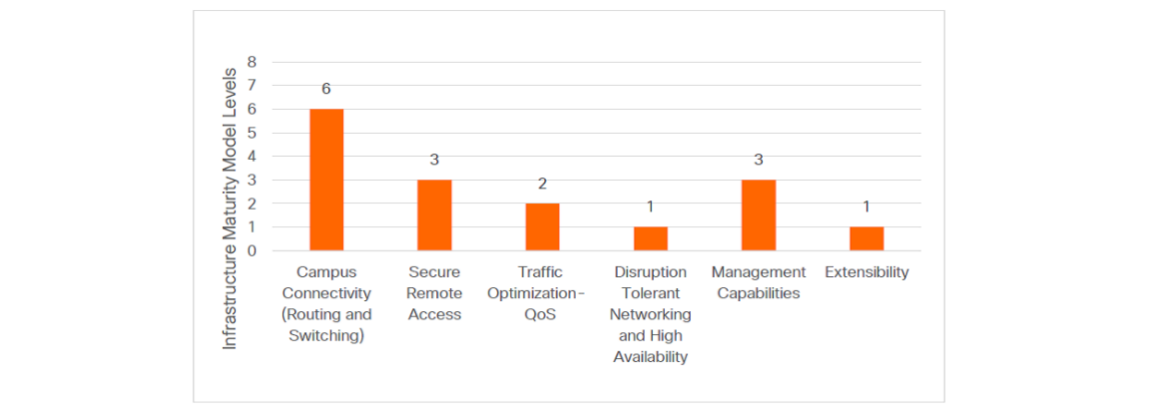
Example of Transport domain assessment output. QoS: quality of service.

As an example, Figure 6 demonstrates a maturity assessment result for the Transport domain of one hospital, showing the individual result for each of the subdomains in the Transport domain.

The potential improvement lies in incrementally improving each subdomain infrastructure capability (as defined in Table 5), thus increasing the value proposition to the next level. Analysis of each subdomain score allows reflection on the current infrastructure capability with the typically experienced limitations highlighted as follows using the Transport example:

- The impact of the subdomain assessment scores needs careful consideration when assessing the value proposition of infrastructure investment. For instance, the high Campus Connectivity score indicates a Transport infrastructure that is approaching high performance capability. However, the lower scores in the associated subdomains indicate potential network unreliability, suboptimal management of data priority, and network failure. This defines an infrastructure that is unable to fully leverage its capabilities.
- While the challenges in Campus Connectivity faced by many hospital facilities are less reflected in this assessment result, challenges commonly include aged infrastructure particularly at the Access layer (5+ years), legacy campus designs that are inflexible, separate physical networks for medical devices and building management systems, a lack of properly defined segmentation policies and design, a lack of network visibility for contextual behavioral analytics, and a lack of API integration capability. These challenges collectively result in a deficiency to support advanced functionality, modularity, and scalability, which is a key requirement to support data use at higher maturity levels.
- The challenges in Traffic Optimization–QoS (a maturity Level 2 in Figure 6) are primarily a result of fragmented QoS designs, the problematic processes to design and implement new campus and wide area network QoS configurations, and no clear roadmap to define an End-to-End contextual Quality of Experience. A lower Traffic Optimization score is often the result of an isolated QoS approach that does not cope well with new services and the associated traffic prioritization requirements.
- Similarly, in the Disruption Tolerant Networking & High Availability subdomain (scoring Level 1 in Figure 6), low scores are often a result of potential for single points of failure in the Core/ Distribution and Access layers of the infrastructure, where devices are locally configured, arising from a lack of comprehensive designs based on Application and Infrastructure interaction.

These challenges provide a snapshot of how the IMA framework can describe the current architecture, identify the misalignment between existing capability and desired capability, and inform subsequent infrastructure planning.

### Framework Extensions

One application of the IMA framework is that it could be mapped to the HIMSS EMRAM, as both frameworks provide a roadmap to support broader process changes in hospitals. Arguably the IMA framework levels could correspond to the application stack requirements to meet the EMRAM model. This would provide a new health care industry benchmark of the maturity of the clinical application deployment and use within hospitals that can be supported by a given level of ICT infrastructure maturity. Localization may be required in applying this framework to countries outside Australia.

### Conclusion

Digital hospitals need to take advantage of the technologies that can improve information flow and use to meet quality clinical and administrative outcomes. The necessity for the technology infrastructure to support these outcomes is clear. Yet such infrastructure is complex and continually evolving in its design and deployment particularly when this involves many stakeholders’ demands and expectations. The resultant assessment of such infrastructure to meet business outcomes and realize value to health care organization through its capability is equally complex.

The use of the Williams CMM Operational Framework allows such capability to be deconstructed into manageable constituent elements and assessed individually. Through this process, it also identifies specific incremental improvement for each capability. The resulting IMA framework allows hospital management and technicians to clearly see how incremental improvements in their infrastructure can be achieved to support clinical and operation goals. Such a method assists hospitals to define an improvement pathway and maturity in delivering their organizational objectives.

The IMA characterizes the technology services required to support a hospital’s information-driven processes. Thus, it provides a tool for determining the preparedness of a hospital facility to support existing or planned process rollouts. The IMA classifies the way hospitals manage their digital infrastructure into an eight-level model that reflects the sophistication of the information processes used within the facility. Each of these levels is characterized in terms of the experiences they generate for the key stakeholders and the technology services required to support those experiences.

Importantly, the IMA framework articulates how hospitals can generate more value from their infrastructure as it defines the levels at which the critical “enabling information” characteristics for an organization are primarily delivered. It describes the stages of information use and resultant clinical/patient experience within a hospital and the ICT infrastructure requirements to reliably achieve those levels of experience. The framework is designed to stage the infrastructure development pathway so that clear benefits can be attributed to the incremental investment that is required to progress from one stage to the next. Consequently, robust business cases can be made for that investment. Ultimately, the purpose of the framework is to map a pathway where Chief Information Officers can see a logical sequence of infrastructure development that they need to take their hospital facility through to reach their desired level of clinical and operational competency.

The development of a generalizable Infrastructure Maturity Assessment tool contributes to and supports the digital hospital industry, providing an international benchmarking standard.

## Appendix

Multimedia Appendix 1 Infrastructure Maturity Assessment framework capabilities.

## Abbreviations

API: application programming interface
CMM: Capability Maturity Model
EMR: electronic medical record
EMRAM: EMR Adoption Model
HIMSS: Healthcare Information and Management Systems Society
ICT: information and communications technology
IMA: Infrastructure Maturity Assessment
IMM: Infrastructure Maturity Model
PAS: patient administration system
QoS: quality of service
REST: Representational State Transfer
SDN: software defined networking
