# Supplementary material for: Improving Digital Hospital Transformation: Development of an Outcomes-Based Infrastructure Maturity Assessment Framework
Source: JMIR Med Inform. 2019 Jan 11;7(1):e12465. doi: 10.2196/12465 (PMC6329893; doi:10.2196/12465)
Supplement: Multimedia Appendix 1 [file medinform_v7i1e12465_app1.pdf]

This is a Multimedia Appendix to a full manuscript published in the J Med Internet Res. For full copyright and citation information see http://dx.doi.org/10.2196/jmir.12465

Infrastructure Maturity Assessment Framework

Domain	Sub-Domain	Capabilities
TRANSPORT	Campus Connectivity	Cabling Standard
		Cabling Design
		Physical Topology Design
		Virtualization
		Access Port Design and Policy
		Core Layer Age of Infrastructure
		Distribution Layer Age of Infrastructure
		Access Layer Age of Infrastructure
		End of Support Status
		Modular Platform Design
		Wired Device Grade
		Access Power Design
		Low-Voltage Power Platform
		Converged Clinical Systems -Wired
		Building Management System (BMS)- Wired
		Software Defined Networking (SDN) Access
		Software Defined Wide Area Networking (SD-WAN)
		Software Defined Controller
		Software Defined Networking (SDN) Integration
	Secure Remote Access	VPN Type
		Secure Remote Access Integration
	Traffic Optimization - QoS	Quality of Service (QoS)
		Quality of Experience (QoE)
	Disruption Tolerant Networking and High Availability	Campus and WAN High Availability
		Disruption Tolerance
		Software Defined Networking (SDN) High Availability
	Management	Network Management
		Software Defined Network (SDN) Management
	Extensibility	IP Addressing Standard
		Extensible IP Address Management Platform
		Extensible Identity Based Segmentation
		Extensible Policy Framework
COLLABORATION	Telephony	Call Control
		Endpoints
		Connectivity
		Architecture
		Integration

		Assurance and Analytics
	Messaging and Presence	Messaging Platform
		Presence Platform
		Clinical Communications Messaging Platform
	Conferencing	Audio Conferencing
		Video Conferencing
		Web Conferencing
		Video Conferencing Endpoints
		Collaboration Experience(s)
	Multimedia Recording and Playback	Multimedia Recording
		Multimedia Storage Compliance
		Multimedia Playback
		Multimedia Recording and Playback Administration and Integration
	Compliance and Security	Session Logging
		Secure Media
		Identity and Access Management (IAM)
	Contact Center	Automatic Call Distribution (ACD) and Queuing
		Call Center Agent Type
		Agent/Supervisor Collaboration
		Campaign
		Contact Center Reporting
		Contact Integration
SECURITY	Network Identity & Access Management	Identity and Access Management (IAM) Solution
		Centralized Authentication and Authorization
		Dynamic Access Policy (DAP)
		Mobile Device Management (MDM) Solution
		Network Admission Control (NAC) posture assessment
		Secure Self Service Portals
		Identity and Access Management (IAM) and Mobile Device Management (MDM) Software Defined Networking (SDN) Integration
	EndPoint Security	Anti-virus and anti-spam on endpoint
		File reputation and analysis
		Heuristics
		Threat analytics and analysis on the endpoint
	Threat Detection & Response	Unified Threat Management (UTM)
		Security Information and Event Manager (SIEM)
		Network Flow Based Anomaly Detection solution
	End Point Posture Validation	Endpoint posture assessment

		Vulnerability analysis result incorporation
		Network device profiling and behaviour analytics to influence network access
		Dynamic remediation
	Firewall/Segmentation Controls	Layer-2/Layer-3 Stateless Access Control Lists (ACL)
		Virtual LAN (VLAN) and Virtual Routing and Forwarding (VRF)
		Firewall
		Application visibility and control
		Dynamic access policies (DAP)
		Application policy model (tag based) in the campus and Data Center based on dynamic attributes/tags learnt from network and endpoint
		Firewall Software Defined Networking (SDN) integration
		Software defined segmentation
	Intrusion Prevention and Threat Visibility	Intrusion Detection/Prevention System (IDS/IPS)
	Anomaly Detection	Data Normalization
		Denial-of-Service (DoS) and Distributed-Denial-of-Service (DDoS) detection and prevention
		Network Behaviour Anomaly Detection (NBAD) solution
		Dynamic Sandboxing (on-premise and in cloud) of unknown files from network and endpoint security systems
		Mobile anomaly detection
	Content Filtering	Web Security and Email security solution
		Spam blocking (text and image)
		Safe unsubscribe
		URL Filtering
		User tracking for education
		DNS-based threat solution
	Encryption	VPN Type
		Disk encryption
	Security Management	Device Policy
		Live Logging
		Centralised Management & Eventing, Event Forwarding
		Advanced Analytics and Correlation
		Security Policy Governed Network
		Security platform SDN integration
	Data Loss Prevention	Data loss prevention strategy
		Data classification
		Network Behaviour Anomaly Detection (NBAD) solution
		Content Data Loss Prevention (DLP) Gateway
		Endpoint Data Loss Prevention (DLP)
	Policy	Security Policy Implementation
		Security Infrastructure Integration

		Security Infrastructure Integration to External Reporting Systems
MOBILITY	Network Access	Wireless Access Point Standard
		Wireless Grade Design
		Location Coverage
		802.11x Wireless Survey
		802.11x Wireless Survey Validation
		802.11x Wireless Age of Infrastructure
		802.11x Wireless End of Support Status
		SSID Access
		Multi Location SSID
		Wi-Fi Quality of Service (QoS)
		WLAN Radio Frequency (RF) Self-Management Design
		Software Defined Networking (SDN) Wireless Access
		Bluetooth Low Energy (BLE) network integrated with the wireless network
		High Availability in Design, including Software Defined Networking (SDN) High Availability)
		Clinical Converged Wireless Network
		Building Management Converged Wireless Network
	Location	Location Services Solution
		Location Analytics (Real-time and Historical Location Analysis) Solution
		Location Accuracy
		Object or Resource Tracking
		Wireless Sensing
		Location Automation
		Navigation
		Service Type
	Device Identity capability including BYOD access	Wireless Identity and Access Management (IAM) Solution
		Wireless Mobile Device Management (MDM) Solution
		Device Ownership
		User Access
		User Role / Identity
		Bring Your Own Device (BYOD)
		Wireless Identity and Access Management (IAM) and Mobile Device Management (MDM) Software Defined Networking (SDN) Integration
	Cellular Network Coverage	Small Cell Coverage (e.g. DAS, Femto/Pico/Metro/Micro Cell) to non-Wi-Fi areas
		Cellular Standard Coverage
DATA CENTER	Network	Dedicated Data Center Network
		Data Center Fabric
	Compute	Access Ports
		Compute Platform Architecture

	Storage	Storage Networking
		Storage Network Connectivity
		Storage Replication Method
		Software Defined Storage (SDS) Provisioning
	Virtualization	Network Virtualization
		Workload Consolidation and Management
		Storage Virtualization
	Automation	Data Center Management Platform
		Data Center Automation
		Data Center Analytics Platform
		Data Center Network Verification Platform
	Cloud	Cloud Strategy
		Public Cloud Usage
		Cloud Orchestration Platform
